# [^18^F]FDG PET/CT Predicts Patient Survival in Patients with Systemic Sclerosis–Associated Interstitial Lung Disease

**DOI:** 10.2967/jnumed.125.269497

**Published:** 2025-07

**Authors:** David M.L. Lilburn, Helen S. Garthwaite, Balaji Ganeshan, Thida Win, Nicholas J. Screaton, Luke R. Hoy, Darren Walls, Raymond Endozo, Robert I. Shortman, Francesco Fraioli, Athol U. Wells, Christopher P. Denton, Ashley M. Groves, Joanna C. Porter

**Affiliations:** 1 Institute of Nuclear Medicine, University College London Hospital, London, United Kingdom;; 2 PET Centre, School of Biomedical Engineering and Imaging Sciences, King’s College London, London, United Kingdom;; 3 Interstitial Lung Disease Service, Department of Respiratory Medicine, University College London Hospital, London, United Kingdom;; 4 Respiratory Medicine Department, Lister Hospital, Stevenage, United Kingdom;; 5 Radiology Department, Papworth Hospital, Cambridge, United Kingdom;; 6 Interstitial Lung Disease Unit, Royal Brompton Hospital, National Heart and Lung Institute, Imperial College, London, United Kingdom;; 7 Centre for Rheumatology, Division of Medicine, University College London, London, United Kingdom; and; 8 UCL Respiratory, University College London and Interstitial Lung Disease Service, University College Hospital, London, United Kingdom

**Keywords:** systemic sclerosis–associated interstitial lung disease, PET/CT, sex-age-physiology score, Kaplan–Meier survival

## Abstract

There are few effective prognostic biomarkers in patients with systemic sclerosis–associated interstitial lung disease (SSc-ILD). We investigated the potential of [^18^F]FDG PET/CT to predict mortality in this population. **Methods:** In total, 45 patients with SSc-ILD (12 men and 33 women; age, 58.9 ± 9.9 y) were prospectively recruited for [^18^F]FDG PET/CT, forming the largest cohort of this type to our knowledge. All patients underwent clinical assessment, including multidisciplinary team review, high-resolution CT evaluation, and pulmonary function tests. The maximum pulmonary uptake on [^18^F]FDG PET/CT (SUV_max_), minimum pulmonary uptake in unaffected or background lung (SUV_min_), and target-to-background ratio (TBR) (SUV_max_/SUV_min_) were quantified using region-of-interest analysis. Kaplan–Meier analysis identified associations with mortality. Associations between [^18^F]FDG PET/CT measurements, pulmonary function tests, and the established model based on sex, age, and lung physiology (known as ILD-GAP) to predict mortality were performed. Stepwise forward Wald–Cox analysis assessed the independence of significant [^18^F]FDG PET/CT measurements from the ILD-GAP index. Synergies between pulmonary [^18^F]FDG PET/CT measurements and ILD-GAP index for risk stratification in patients with SSc-ILD were investigated. **Results:** Forty-five patients with SSc-ILD were followed for a mean of 44.8 ± 26.1 mo, with 15 deaths (33%) recorded. The mean ± SD SUV_max_ was 3.2 ± 1.1, SUV_min_ was 0.5 ± 0.3, and TBR was 6.8 ± 2.6. Increased mortality was associated with high pulmonary SUV_max_ (*P* = 0.027), high SUV_min_ (*P* = 0.002), low TBR (*P* = 0.016), low forced vital capacity (*P* = 0.021), low carbon monoxide diffusion coefficient (*P* = 0.021), low transfer factor (*P* = 0.012), high ILD-GAP score (*P* = 0.010), and high ILD-GAP index (*P* = 0.005). Multivariate Cox regression analysis revealed that pulmonary SUV_min_ (hazard ratio, 4.2; 95% CI, 1.3–13.4; *P* = 0.017) and ILD-GAP index (hazard ratio, 3.9; 95% CI, 1.2–12.8; *P* = 0.024) were the only independent predictors of overall survival. Combining [^18^F]FDG uptake with ILD-GAP score data in a modified ILD-GAP index refined the ability to predict mortality (*P* < 0.002). **Conclusion:** High-background [^18^F]FDG uptake in normal-appearing lung independently predicts overall survival in SSc-ILD and may stratify patients’ risk when combined with ILD-GAP score data in a modified ILD-GAP index. High pulmonary [^18^F]FDG uptake is associated with increased mortality in patients with SSc-ILD.

## Visual Abstract

**Figure:**
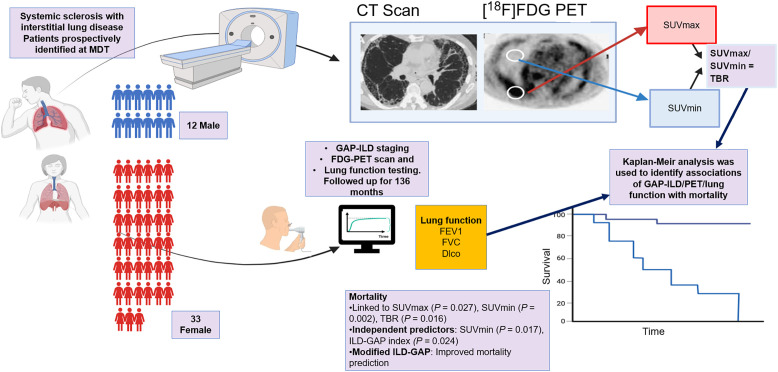

Systemic sclerosis (SSc) is a chronic, autoimmune rheumatic disease characterized by skin thickening and organ fibrosis, often involving the lungs (1). The etiology is unknown, but genetic and environmental factors are implicated, and the disease is more prevalent in women. SSc is a heterogeneous disease (2) and involves immune dysregulation, endothelial dysfunction, and excess extracellular matrix deposition by fibroblasts, leading to fibrosis.

Complications such as interstitial lung disease (ILD) and pulmonary vascular disease are the main cause of death in patients with SSc (3). ILD severity ranges from mild, limited interstitial involvement to rapidly progressive fibrotic disease and respiratory failure (4). Predictors of progressive ILD include a shorter interval between onset of skin disease and pulmonary fibrosis, male sex, Black race, and concomitant cardiac disease.

High-resolution CT (HRCT) acts as a surrogate for histologic diagnosis of SSc-ILD. HRCT findings range from inflammatory, nonspecific interstitial pneumonia to more fibrotic usual interstitial pneumonia (5). In general, outcomes and treatment response correlate with the extent of lung involvement, with worse outcomes associated with usual interstitial pneumonia.

Treatment approaches rely on symptom monitoring, pulmonary function tests (PFTs), and HRCT but lack dynamic disease assessment and progression risk. Functional measurements may be confounded by the multisystem nature of SSc-ILD (6), with cardiovascular, cutaneous, and musculoskeletal disease manifestations affecting performance during exercise testing. PFTs are often insensitive to early lung disease (7) and affected by patient compliance and pulmonary arterial hypertension (8). New immunosuppressive regimens and antifibrotic agents demand new prognostic biomarkers to predict outcome and treatment response in patients with SSc-ILD.

PET/CT can noninvasively investigate cellular metabolism in vivo, and [^18^F]FDG PET/CT is emerging as a potential biomarker in idiopathic pulmonary fibrosis (IPF) (9–11), aiding patient stratification (12–14). Small retrospective studies have explored [^18^F]FDG PET/CT associations in SSc-ILD (15–19), and a prospective study of 23 patients has attempted to define a link with serum biomarkers in SSc-ILD (20).

In this study, we compared the use of [^18^F]FDG PET/CT imaging with prognostic scores obtained from a prediction model based on sex, age, and lung physiology (known as ILD-GAP) (21) to predict mortality in patients with SSc-ILD.

## MATERIALS AND METHODS

**FIGURE 1.**
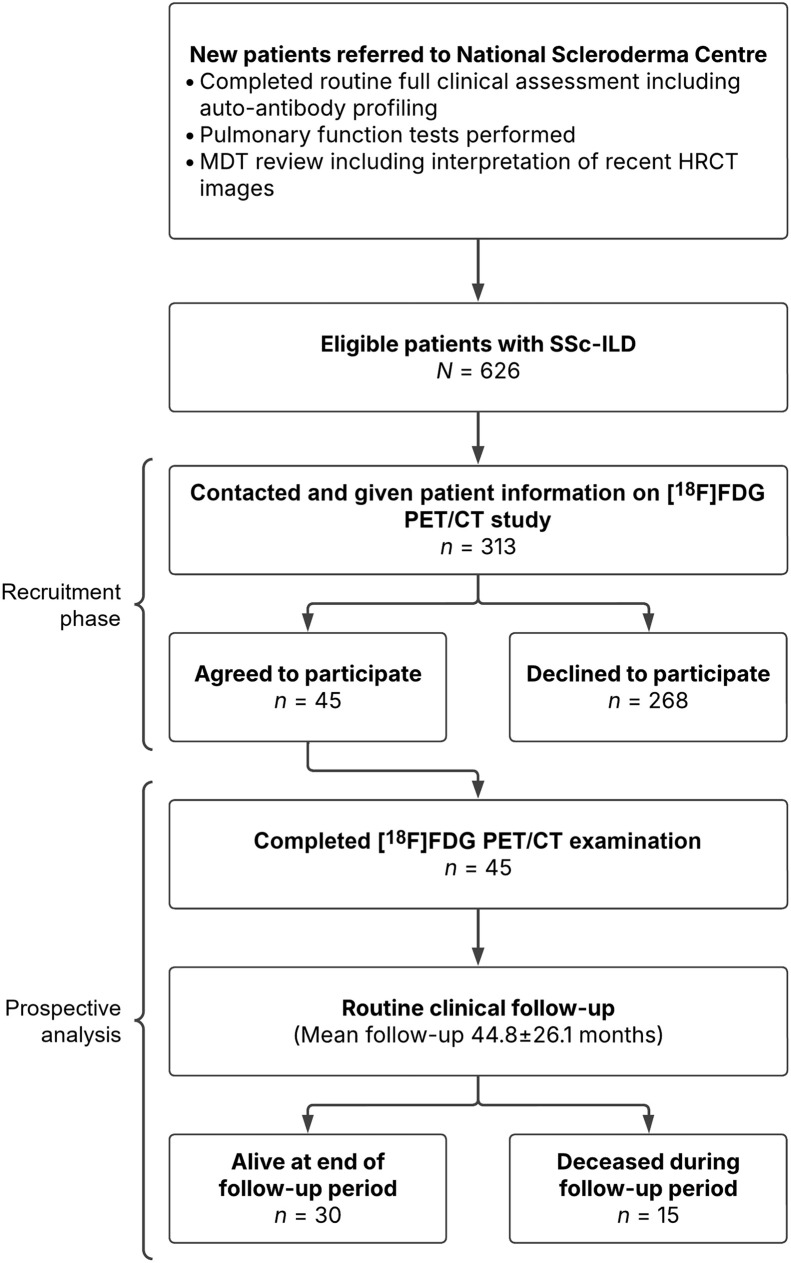
Flow diagram detailing study recruitment and analysis phases. MDT = multidisciplinary team.

This prospective study and its protocol were approved by the London-Harrow Research Ethics Committee, and all participants signed a written, informed consent form. Potential subjects were identified from a population of 626 patients referred to the National Scleroderma Centre, Royal Free Hospital. In total, 313 patients were approached, and 45 agreed to participate and were consecutively recruited from April 2010 to June 2018 (12 men and 33 women; age, 58.9 ± 9.9 y). All 45 underwent [^18^F]FDG PET/CT at the Institute of Nuclear Medicine, University College London Hospital. Patients were diagnosed with SSc-ILD after multidisciplinary team review, HRCT, and PFTs, including forced vital capacity (FVC), forced expiratory volume in 1 s (FEV_1_), and carbon monoxide transfer factor and coefficient (TL_CO_/K_CO_). Patients with clinical or radiologic suspicion of infection or neoplasia were excluded. The recruitment process and observational phases are detailed in Figure 1.

### PET/CT Image Acquisition

[^18^F]FDG PET/CT imaging of the thorax was performed after diagnosis. All images were acquired on the same PET/CT scanner (VCT PET/64-detector CT instrument; GE HealthCare). Patients were injected with a mean 197.9 ± 18.4 MBq of [^18^F]FDG. After 1 h of uptake, patients were positioned supine on the CT table with their arms held above their heads and instructed to remain still. A CT scan was performed for attenuation correction and PET coregistration purposes, immediately followed by a [^18^F]FDG PET emission scan with identical anatomic coverage (8 min per bed position).

### Image Analysis

#### Observers

[^18^F]FDG PET/CT images were analyzed by a PET radiologist and a PET technologist with more than 10 y of experience quantifying pulmonary uptake in [^18^F]FDG PET/CT imaging of SSc-ILD, under the supervision of a senior radiologist or nuclear medicine physician. CT/HRCT imaging was reviewed by a thoracic radiologist with expertise in ILD.

#### Image Display and Processing

**FIGURE 2.**
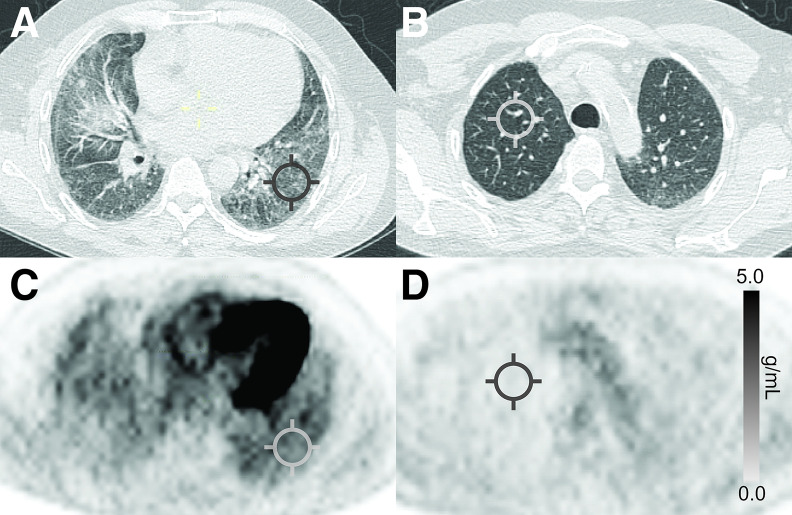
Axial CT and corresponding [^18^F]FDG PET emission scans of patient with nonspecific interstitial pneumonia. CT placement of regions of interest on morphologically abnormal (A) and normal (B) lung. Corresponding regions of interest on [^18^F]FDG PET emission scan for determining SUV_max_ (C) and SUV_min _(D).

Quantification of [^18^F]FDG PET/CT metrics were performed on a propriety workstation through placement of small, 2-dimensional regions of interest on left and right lung fields on coregistered axial images. SUV_max_ was calculated as the highest single pixel value within a region of interest drawn over the area of most intense pulmonary [^18^F]FDG uptake (Fig. 2). The SUV_min_ was calculated as the lowest single pixel value within a region of interest drawn over normal-appearing lung parenchyma exhibiting the lowest pulmonary [^18^F]FDG uptake. Normal-appearing lung was confirmed by the thoracic radiologist with reference to prior HRCT imaging. SUV_min_ was considered a measure of the background lung uptake and used to calculate the target-to-background ratio (TBR = SUV_max_/SUV_min_) (9,14).

A thoracic radiologist masked to the PET emission scans classified ILD abnormalities as either usual interstitial pneumonia or nonspecific interstitial pneumonia based on CT/HRCT images, as previously described (9,14).

### ILD-GAP Calculation

ILD-GAP score was calculated using 4 variables: sex, age, FVC, and TL_CO_ (21). The calculated score falls within a continuous range from 0 to 8, with higher scores indicating poorer outcomes. The score translates to an ILD-GAP index of I–IV, corresponding to scores of 0 or 1, 2 or 3, 4 or 5, and 6–8, respectively.

### Patient Follow-up

The follow-up period was defined as the period between the date of the patient’s [^18^F]FDG PET/CT scan and the date of the patient’s death or August 2021 (11 y 4 mo after study initiation). Patient survival was confirmed using patient charts, electronic databases, primary health care records, and telephone interviews.

### Statistical Analysis

Statistical analyses were performed using SPSS for Windows version 19.0 (IBM). Data were reported as mean ± SD. A *P* value of less than 0.05 was considered statistically significant.

#### Univariate Survival Analysis

Relationships between [^18^F]FDG PET/CT parameters, PFTs, and ILD-GAP scores and indices and patient survival were assessed using univariate Kaplan–Meier survival analysis. Differences in the survival plots were evaluated using a nonparametric log-rank test. The optimized parameter value (corresponding to the lowest *P* value from log-rank test) was used as a cutoff to separate the survival plots into poorer and favorable prognoses groups. Kaplan–Meier curves for each group illustrate the proportion of patients surviving at a given time.

#### Multivariate Cox Regression Analysis

Cox proportional hazards regression test assessed time independence of the significant univariate markers. Stepwise forward Wald regression method was used to identify which significant parameters were independent predictors of survival.

#### Modeling PET Data with ILD-GAP Analysis

ILD-GAP scores were combined with individual [^18^F]FDG PET/CT parameters SUV_max_, SUV_min_, and TBR to create modified ILD-GAP calculations (mGAP) to determine whether they could improve the ILD-GAP scores’ ability to predict survival (14).

[^18^F]FDG PET/CT parameters were incorporated into the ILD-GAP score and coded as a 1 or 0, reflecting a poor or favorable prognosis, respectively (defined by the optimized cutoff). The resulting mGAP score ranged from 0 to 9, and the mGAP index remained I–IV, corresponding to mGAP scores of 0–2, 3 or 4, 5 or 6, and 7–9, respectively.

## RESULTS

Forty-five patients with SSc-ILD were followed for a mean of 44.8 ± 26.1 mo, with 15 deaths (33%) recorded. All patients provided measurements of FEV_1_ and FVC, but 10 were unable to complete tests to measure TL_CO_ and K_CO_. Forty-two patients (93%) had predominantly nonspecific interstitial pneumonia, and the remaining 3 patients (7%) displayed a usual interstitial pneumonia pattern. Patient characteristics are summarized in Table 1.

**TABLE 1.** Patient Characteristics at Baseline with PFT and CT Findings, *n* = 45

Characteristic	Value
Clinical data*	
Age (y)	58.9 ± 8.9
Female	33 (73)
Male	12 (27)
PFT^†^	
Predicted FVC (%)	70 (59–86.5)
Predicted FEV_1_ (%)	667 (57.5–84)
K_CO _(min^−1^)	66 (30.5–85)^‡^
TL_CO_ (mmol min^−1^ kPa^−1^)	41 (25–57)^‡^
Autoantibodies*	
Antiscleroderma-70	19 (42)
Anticentromere antibody	4 (9)
Anti-RNA-polymerase III	2 (4)
Other^§^	9 (20)
No specific antibodies	10 (22)
CT pattern*	
NSIP	42 (93)
UIP	3 (7)

* Continuous variables are expressed as mean ± SD. Qualitative variables are expressed as number with percentages in parentheses.

† Data expressed as median with interquartile range in parentheses.

‡ *n* = 35; 10 patients were unable to complete TLCO and KCO tests.

§ Includes U1 ribonucleoprotein and rare antinuclear antibodies.

NSIP = nonspecific interstitial pneumonia; UIP = usual interstitial pneumonia.

**FIGURE 3.**
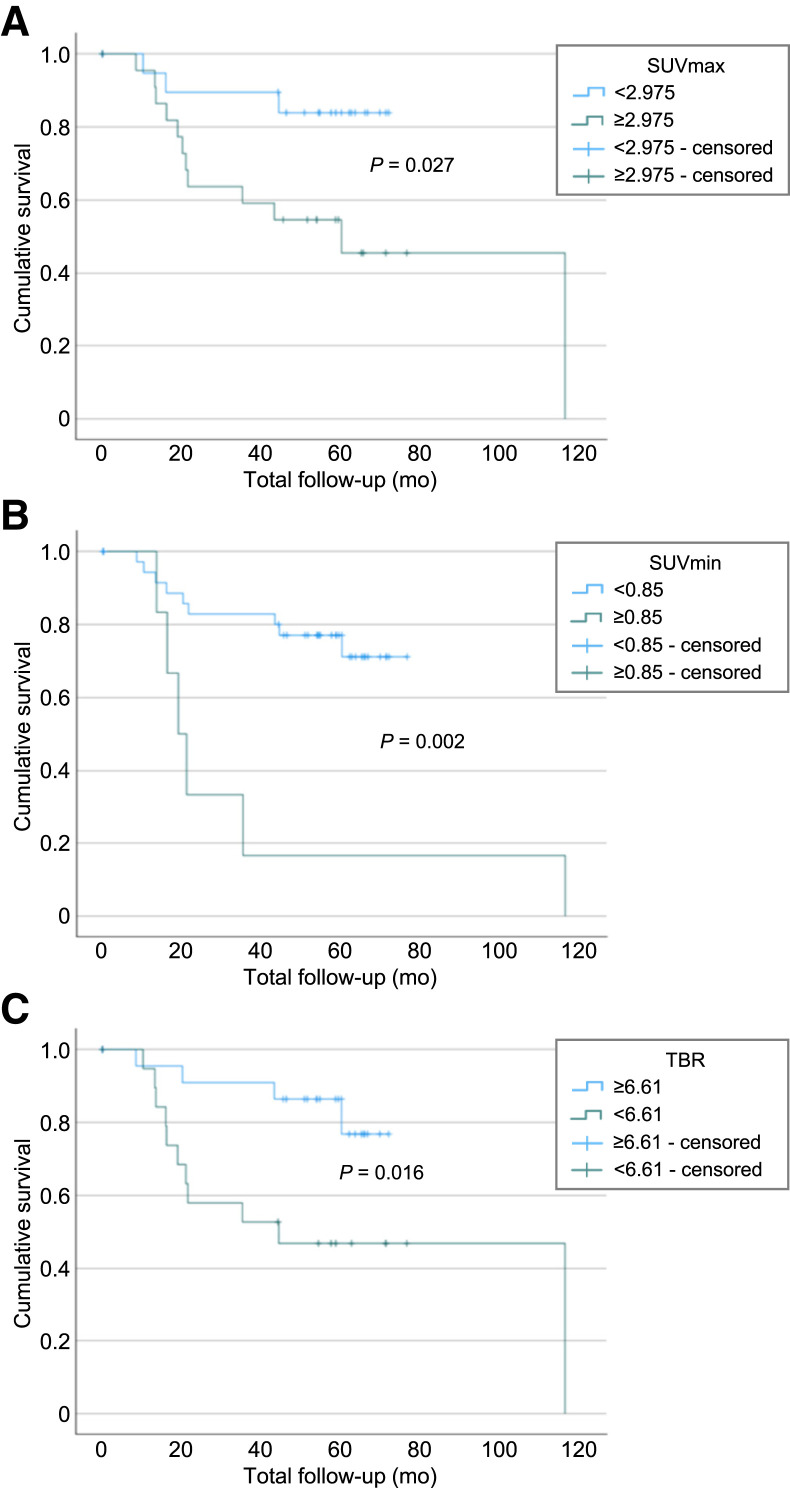
Kaplan–Meier survival curves for SUV_max_ (cutoff value, 2.975) (A), SUV_min_ (cutoff value, 0.85) (B), and TBR (cutoff value, 6.61) (C).

Table 2 summarizes SUV_max_, SUV_min_, and TBR values and significant associations between [^18^F]FDG uptake (with optimized cutoffs) and survival. Figure 3 displays associated Kaplan–Meier survival curves.

**TABLE 2.** Kaplan–Meier Survival Analysis Based on Optimized Cutoff Values for PET and PFT Parameters and ILD-GAP Scores and Indices

			Poorer prognosis group		Favorable prognosis group	
PET/PFT marker	Mean ± SD	Optimized cutoff for poor survival	Number of patients	Survival (mo)	Number of patients	*P*
SUV_max_	3.2 ±1.2	≥2.975	23	60.1	22*	0.027
SUV_min_	0.5 ±0.3	≥0.85	6	18.9	39*	0.002
TBR	6.8 ±2.6	<6.61	20	44.3	25*	0.016
FVC (%)	72.0 ±19.7	<72.5	25	60.1	20*	0.021
FEV_1_	69.0 ±18.2	<65.5	20	60.1	25*	0.142
TL_CO_	47.8 ±18.4^†^	<55.5	32	116.3	13*	0.012
K_CO_	75.1 ±20.7^†^	<63.5	20	43.2	25 (116.3^‡^)	0.021
ILD-GAP score	1 (0–2)^§^	≥1	26	43.2	19*	0.010
ILD-GAP index	II (I–III)^§^	≥II	19	21.4	26 (116.3^‡^)	0.005

* Median survival not calculated.

† 10 patients were unable to complete gas transfer measurement of TL_CO_/K_CO_, and these were classified as poor values (i.e., below median). Mean ± SD calculated with *n* of 35.

‡ Number in parentheses is survival in months.

§ Data expressed as median with interquartile ranges, given discrete values.

For patients whose SUV_max_ was 2.975 or greater, 2- and 5-y survival rates were approximately 64% and 46%, respectively, with 50% mortality within 60 mo. For those whose SUV_max_ was less than 2.975, 2- and 5-y survival rates increased to 90% and 84%, respectively, and 50% mortality was not achieved. When the TBR did not exceed 6.61, 2- and 5-y survival rates were approximately 60% and 50%, respectively, with 50% mortality within 44 mo. When the TBR exceeded 6.61, 2- and 5-y survival rates increased to approximately 91% and 77%, respectively, and 50% mortality was not achieved.

Median SUV_min_ did not significantly inform outcomes, therefore a cutoff of at least 0.85 was determined. Of the 6 patients with an SUV_min _of at least 0.85, 2- and 5-y survival rates were 34% and 17%, respectively, with a 50% mortality within 19 mo. Below this threshold, 2- and 5-y survival rates were 83% and 71%, respectively, and 50% mortality was not achieved.

**FIGURE 4.**
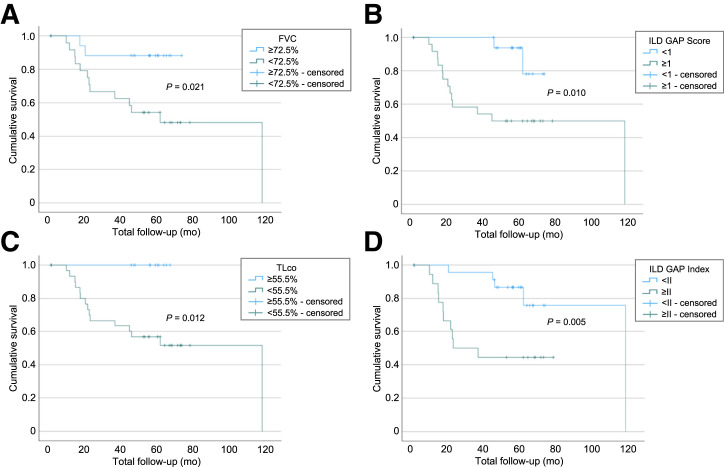
Kaplan–Meier survival curves for FVC (cutoff value 72.5%) (A), ILD-GAP score (cutoff value, 1) (B), TL_CO _mmol min^−1^ kPa^−1^ (cutoff value, 55%) (C), and ILD-GAP index (cutoff value, II) (D).

Survival was significantly associated with FVC, TL_CO_, K_CO_, and ILD-GAP scores and indices. The 2- and 5-y survival rates and median survival values are shown in Table 3 and Figure 4.

**TABLE 3.** Estimated 2- and 5-Year Survival Rates Based on Optimized Cutoff Values for PET and PFT Parameters and ILD-GAP Scores and Indices

Variable	SUV_max_	SUV_min_	TBR	ILD-GAP score	ILD-GAP index	FVC	TL_CO_	K_CO_
Optimized cutoff	2.975	0.85	6.61	1	II	72.5%	55.5%	63.5
2-y survival above cutoff	64%	34%	91%	59%	50%	88%	100%	95%
5-y survival above cutoff	46%	17%	77%	50%	45%	88%	100%	75%
2-y survival below cutoff	90%	83%	58%	100%	96%	67%	67%	55%
5-y survival below cutoff	84%	71%	47%	78%	76%	48%	52%	50%
50% mortality above threshold*	60	19	NA	43	21	NA	NA	116
50% mortality below threshold*	NA	NA	44	NA	116	60	116	43

* Estimate of median survival where applicable in months (median survival was not calculated when 50% mortality was not achieved).

NA = not applicable.

### Multivariate Cox Regression Analyses

Cox proportional hazards regression test demonstrated time independence for all significant univariate markers, meeting the proportional hazards assumption. SUV_min _was found to be independent of ILD-GAP values for predicting prognoses. When SUV_min_ and ILD-GAP index were included in the Cox regression model, SUV_min_ (threshold > 0.85; hazard ratio, 4.2; 95% CI, 1.3–13.4; *P* = 0.017) and ILD-GAP index (threshold > 1.5; hazard ratio, 3.9; 95% CI, 1.2–12.8; *P* = 0.024) were independent predictors of survival.

### Modeling of PET-Derived SUV_min_ in Combined ILD-GAP Analysis

**FIGURE 5.**
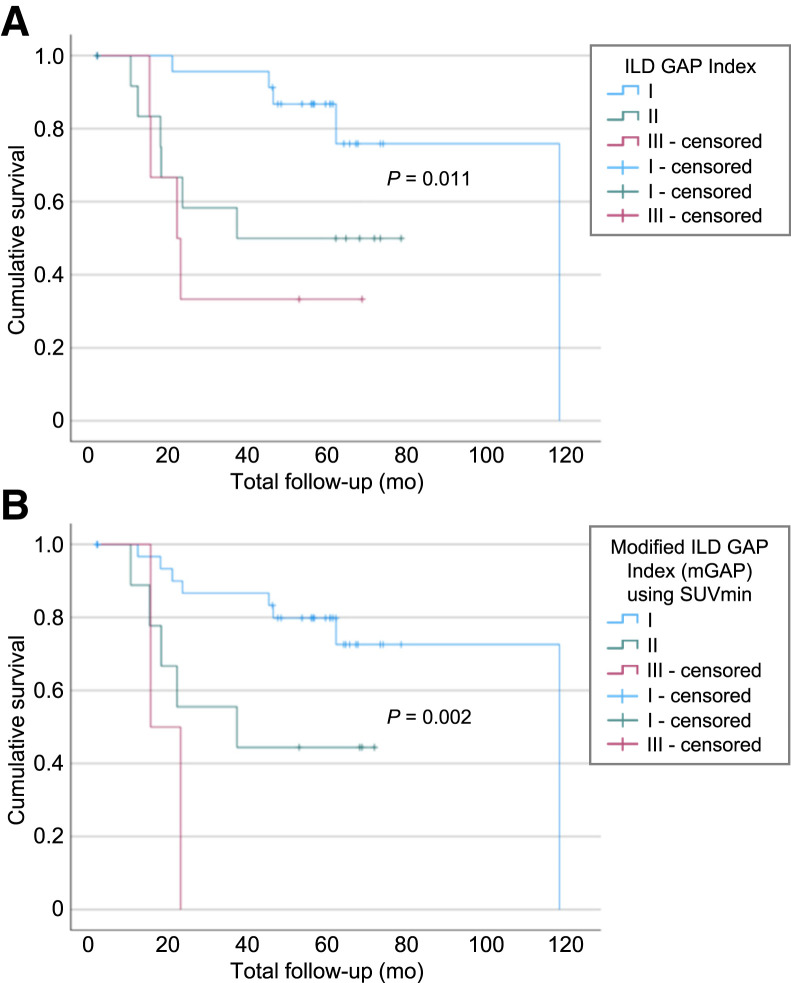
Kaplan–Meier survival curve for ILD-GAP index (A) and modified ILD-GAP index incorporating SUV_min_ (B).

There was synergy in survival associations between SUV_min_ and ILD-GAP index with mGAP using SUV_min_, showing improved risk stratification over the original ILD-GAP index (Fig. 5).

## DISCUSSION

We present the largest cohort to date, to our knowledge, of prospective [^18^F]FDG PET/CT data for patients with SSc-ILD. We have shown that baseline measurements of [^18^F]FDG uptake correlate with patient survival. High SUV_max_, high SUV_min_, and low TBR were associated with poorer survival, but SUV_min_ had an independent prognostic benefit to the ILD-GAP index. SUV_min_ may be particularly helpful in prognosticating SSc-ILD because of its potential to reflect underlying diffuse metabolic activity in lung tissue that appears normal on conventional imaging. Unlike SUV_max_, which highlights focal areas of intense metabolic activity often linked to severe inflammation or fibrosis, SUV_min_ captures the lowest level of [^18^F]FDG uptake within the lung. This measure may be indicative of global lung involvement, potentially reflecting subclinical inflammation, early fibrotic changes, or metabolic alterations that are not yet structurally apparent. In the context of SSc-ILD, where disease progression often occurs diffusely rather than focally, SUV_min_ might serve as a surrogate for widespread, low-grade inflammatory processes that contribute to overall lung function decline. Additionally, SUV_min_’s independent prognostic benefit over the ILD-GAP index suggests that it provides unique biologic insights beyond traditional clinical parameters. This aligns with previous findings that [^18^F]FDG uptake in seemingly normal lung parenchyma was linked to poor outcomes in ILD, reinforcing the notion that metabolic alterations may precede structural damage (10,12). However, the exact mechanisms underlying SUV_min_’s prognostic value remain unclear. It could relate to differences in baseline lung metabolism, microvascular dysfunction, or early fibrotic activity that is not yet radiographically evident. Further studies with larger, independent cohorts are necessary to validate these findings and elucidate the biologic significance of SUV_min_ in SSc-ILD progression.

FVC, TL_CO_, and K_CO_ in combination with sex and age in ILD-GAP scores and indices were predictors of survival. PFTs are often challenging for patients with SSc because limited respiratory movements are a feature of advanced disease (10 patients in our cohort were unable to perform tests to obtain TL_CO _and K_CO_ values). TL_CO_ is a composite marker of several physiologic processes and is reduced in the presence of pulmonary arterial hypertension (22), where increased pulmonary [^18^F]FDG uptake may also be seen (23,24). It is unclear if this was a factor in our study.

Although SUV_max_ and TBR were not independent of the ILD-GAP index, they were prognostic and might be useful in patients for whom PFTs are impossible, with TBR having the advantage of normalizing glucose uptake to background activity.

Attempts to achieve consensus around imaging data analysis methods for pulmonary [^18^F]FDG PET/CT in lung disease include the use of TBR as a quantitative biomarker in the monitoring of ILD (25). In contrast to this study, previous research has shown that high pulmonary TBR is independently associated with increased mortality among patients with IPF (14). This discrepancy may reflect differences in the relative contributions of inflammation and fibrosis to [^18^F]FDG PET/CT signal. Nonspecific interstitial pneumonia is predominant in SSc-ILD, with inflammation being the primary driver, whereas fibrosis is predominant in IPF. The precise cellular mechanisms underlying [^18^F]FDG PET/CT signal in SSc-ILD and IPF are unknown, although early data in fibrotic ILD suggests that [^18^F]FDG PET/CT signal correlates with neoangiogenesis (26). The radiopharmaceutical ^68^Ga-labeled fibroblast activation protein inhibitor 4 binds to fibroblast activation protein and has been proposed as a biomarker in SSc-ILD (27), although how it relates to [^18^F]FDG uptake is unknown. Investigations into survival predictors in SSc-ILD typically involve low numbers of patients due to the low incidence of the disease. Patients with an FVC exceeding 70% of the predicted value are considered to have limited disease, even with 30% lung involvement detected by HRCT (28,29). An analysis of data from the SENSCIS study, investigating the efficacy and safety of nintedanib, noted that a decrease in FVC% predicted of 3% or greater was associated with earlier initial hospitalization or death during 52-wk follow-up (30). It is debatable if this would be detectable outside of a clinical trial, where progression is often defined as an FVC reduction of more than 10% or a TL_CO _reduction of more than 15% (31).

The addition of FVC and TL_CO _into scoring systems (including ILD-GAP) requires patients to complete both tests, and test intervals of 12–24 mo are often necessary to identify meaningful changes. The intention of the ILD-GAP score was patient stratification rather than individual outcome prediction; however, in the era of precision medicine, the incorporation of [^18^F]FDG PET/CT into the calculus may bridge that gap.

Mortality prediction is important for shared decision-making, including referral for hematopoietic stem cell or lung transplantation. We have shown that high [^18^F]FDG uptake in normal-appearing lung (SUV_min_) in SSc-ILD correlates with increased mortality and provided further evidence that high SUV_max_ almost doubles the risk of death in patients with SSc-ILD.

Presently, limited or stable SSc-ILD is not considered eligible for treatment with first-line mycophenolate mofetil or cyclophosphamide (32). Second-line treatment with immune modulators, such as tocilizumab or rituximab, is usually reserved for patients who do not respond to initial treatment. This observation is based on PFT and HRCT surveillance and can take 6–12 mo to become appreciable. Finally, given the associated toxicities, antifibrotic drugs are often reserved for progressive fibrotic disease. The overall mortality in this cohort was higher than that of recent clinical trials, indicating that high pulmonary [^18^F]FDG uptake may identify patients with poorer survival independent of PFT values. This raises the possibility of selecting patients for escalated immune modulation from a wider subpopulation or potentially using pulmonary [^18^F]FDG uptake as a response biomarker in drug development.

Interest in models of risk prediction incorporating clinical and imaging parameters is growing, particularly with IPF, although the GAP model has been modified for use across all ILDs, including SSc-ILD, and has performed well (21,33,34). These models are most useful when stratifying patients for clinical trials rather than assessing individuals. Serum biomarkers may identify patients at risk of disease progression, and future studies combining clinical models, serum biomarkers, and functional imaging may refine the theranostic approach in SSc-ILD.

HRCT is the main diagnostic imaging tool for SSc-ILD. Several studies have provided associations between survival data and scoring and grading systems of HRCT findings (35), but data showing advantages of this imaging technique in clinical trials are limited. In contrast, our imaging approach uses metabolic functional data and the exquisite sensitivity of [^18^F]FDG PET/CT.

Increased [^18^F]FDG uptake is seen in morphologically normal lung parenchyma in IPF (10,12) and in small cohorts of patients with SSc-ILD (13,15,17,19), although this has been an inconsistent finding, with 2 recent, larger studies reporting no increased [^18^F]FDG uptake in normal-appearing lung (18,20). None of the prior, smaller studies of [^18^F]FDG PET/CT in these patients found a link between uptake values and mortality, although most found associations with PFT values. Whether this is related to study size, differing acquisition and analysis methodologies, or the patient populations is unclear.

This study was limited by the small size of the cohort but is the largest of its type combining PFTs with [^18^F]FDG PET/CT datasets. To increase recruitment, the [^18^F]FDG PET/CT was not always performed at diagnosis, but our data imply that prognosis may be determined at various stages of disease. Technical factors, such as variations around radiopharmaceutical dose and uptake time, respiratory gating, more complex air-fraction correction, and compartmental modeling approaches, require consideration. The techniques used here acknowledge the challenges of such imaging, with the methods recognized as robust (25). We have been able to make significant survival observations using these routine [^18^F]FDG PET/CT measurements.

## CONCLUSION

High [^18^F]FDG uptake in background, normal-appearing lung positively correlated with mortality and can combine with ILD-GAP index for improved prognostication as a clinically needed biomarker to stratify patients’ risk of SSc-ILD. We present further evidence that high [^18^F]FDG uptake positively correlates with mortality.

## DISCLOSURE

The Institute of Nuclear Medicine receives funding for idiopathic pulmonary fibrosis research from GSK (CRT115549) Research and Development, Stevenage, U.K. This work was undertaken at UCLH/UCL, which received a proportion of the funding from the U.K.’s Department of Health’s NIHR Biomedical Research Centre’s funding scheme. No other potential conflict of interest relevant to this article was reported.

## Notes

KEY POINTS **QUESTION:** Can [^18^F]FDG PET/CT metrics improve the ability of the ILD-GAP score to predict survival in patients with SSc-ILD? **PERTINENT FINDINGS:** This prospective, cohort study recorded [^18^F]FDG PET/CT and PFT metrics in 45 patients with SSc-ILD and found that high pulmonary [^18^F]FDG uptake in morphologically normal lung tissue (SUV_min_) was independently associated with survival (*P* = 0.027). SUV_min_ enhanced the prognostic value of the ILD-GAP score when incorporated into a modified ILD-GAP index, significantly improving risk stratification (log-rank text, *P* = 0.002). Additionally, elevated SUV_max_ was associated with increased mortality. **IMPLICATIONS FOR PATIENT CARE:** [^18^F]FDG PET/CT may provide a clinically needed biomarker to stratify patients’ risk and facilitate prompt access to second-line treatments for patients with SSc-ILD.
